# ﻿Unveiling the hidden diversity: the second species of *Wenchengia* (Lamiaceae) discovered from a dry mountaintop meadow of Hainan Island, China

**DOI:** 10.3897/phytokeys.259.155679

**Published:** 2025-07-08

**Authors:** Si-Cheng Xu, Jin-Fei Xiao, Chun-Lei Xiang, Bo Li

**Affiliations:** 1 Center for Integrative Conservation, Xishuangbanna Tropical Botanical Garden, Chinese Academy of Sciences, Mengla 666303, China Chinese Academy of Sciences Mengla China; 2 CAS Key Laboratory for Plant Diversity and Biogeography of East Asia, Kunming Institute of Botany, Chinese Academy of Sciences, Kunming 650201, China Chinese Academy of Sciences Kunming China; 3 State Key Laboratory of Phytochemistry and Natural Medicines, Kunming 650201, China State Key Laboratory of Phytochemistry and Natural Medicines Kunming China

**Keywords:** Endangered species, Lamiales, morphology, new taxa, Scutellarioideae

## Abstract

*Wenchengiawui*, a remarkable new species of *Wenchengia* endemic to a dry mountaintop meadow of Sanya City, Hainan Province, southern China, is described and illustrated. The new species is readily distinguished from the only other extant species, *W.alternifolia*, by its opposite phyllotaxy, oblong-ovate leaves with rounded to slightly truncate bases, shallowly funnelform calyces with spreading lips, and 8-ribbed nutlets, as well as its unique habitat preference. The new species is categorized as Critically Endangered (CR) according to the IUCN Red List criteria, based on its limited distribution, specialized habitat, and small population size. Detailed information on its distribution, habitat, diagnostic characteristics, and a comprehensive morphological description are provided. The discovery of this new species offers valuable insights into the evolutionary history and conservation strategies of *Wenchengia*.

## ﻿Introduction

*Wenchengia* C.Y.Wu & S.Chow is a previously monotypic genus within Scutellarioideae of Lamiaceae, along with other five genera: *Holmskioldia* Retz., *Renschia* Vatke, *Scutellaria* L., *Tinnea* Kotschy ex Hook.f., and the recently described *Heliacria* Bo Li, C.L.Xiang, T.S.Hoang & Nuraliev (Li et al. 2023). *Wenchengia* and its sole species *W.alternifolia* C.Y.Wu & S.Chow was initially described by Wu and Chow (1965), based on two collections in the 1930s. Due to its distinctive morphological features, i.e., alternate phyllotaxy, raceme-like inflorescence, and nutlets connected with receptacle by slander stalks, the genus *Wenchengia* was placed in its own subfamily Wenchengioideae (Wu and Chow 1965; Li et al. 2012). Although morphological, anatomical, and cytological data suggested a close relationship of *Wenchengia* with Scutellarioideae or Ajugoideae (Wu and Chow 1965; Cantino and Abu-Asab 1993; Ryding 1996), its precise phylogenetic placement remained unresolved due to the absence of living materials.

Harley et al. (2004) reported that *Wenchengiaalternifolia* had not been found in the wild since its description and was even presumed extinct. Later in 2010, a living population of *W.alternifolia* was rediscovered at the type locality in Wanning County, Hainan Province. Subsequent phylogenetic analyses based on *rbcL* and *ndhF* sequences confirmed *Wenchengia* as an early-diverging and relictual lineage of Scutellarioideae (Li et al. 2012), which was further proved by plastome data (Zhao et al. 2020, 2021). While *Wenchengia* was long believed to be endemic to Hainan Island, specimens from Vietnam were found by Paton et al. (2016), followed by the discovery of extant populations from Vietnam by Bo Li and his team in 2017 (Zhou et al. 2022). Further expanding its known range, Chunlei Xiang discovered an additional population at Ding’an County of Hainan (Zhou et al. 2022), suggesting a broader distribution and potential species diversity within the genus than previously recognized.

**Figure 1.**
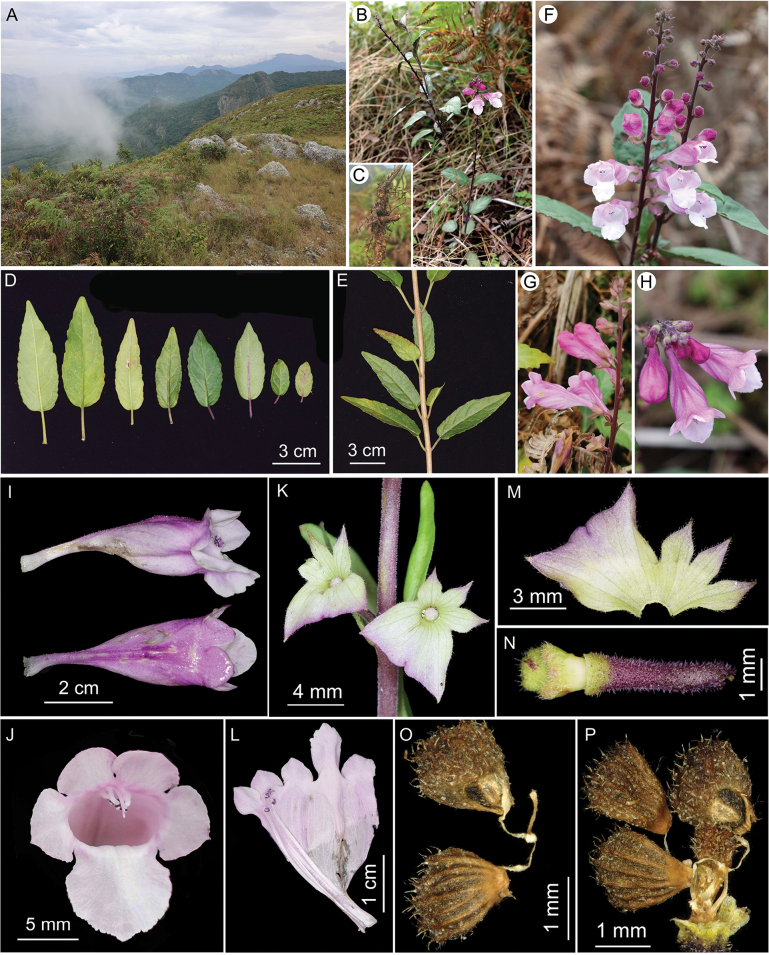
*Wenchengiawui* sp. nov. **A.** Habitat; **B.** Habit; **C.** Rootstock; **D.** Leaves; **E.** Leafy stem, showing its opposite phyllotaxy; **F.** Inflorescences; **G, H.** Flowers; **I.** Lateral and dorsal views of corolla; **J.** Top view of corolla; **K.** Top view of calyces; **L.** Dissected corolla; **M.** Dissected calyx; **N.** Lateral view of ovary and pedicel; **O, P.** Nutlets connected by stalks.

In July 2023, an unusual population of *Wenchengia* was discovered at a mountaintop of Baishiling Mountain in Tianya District, Sanya City, Hainan Province. Unlike all previously known populations of *W.alternifolia*, which are characterized by alternate phyllotaxy and typically inhabit moist, shaded streamside environments within rainforests (Wu and Chow 1965; Wu and Li 1977; Li et al. 2012), this recently discovered population grows in a dry montane meadow (Fig. 1A) and exhibits opposite phyllotaxy, differing significantly from the extant species. A detailed field investigation in October revealed additional differences in the leaf shape, calyx structure, and nutlet morphology (Fig. 1). Based on comprehensive morphological comparisons, literature review, and herbarium specimen examinations, we recognize it as a new species, *W.wui* Bo Li, S.C.Xu & C.L.Xiang, as described below.

## ﻿Material and methods

Field investigations were conducted in October 2023 on Baishiling Mountain, located in Tianya District, Sanya City, Hainan Province, and voucher specimens bearing both flowers and fruits were collected. Morphological description of the putative new species was based on observations of living individuals in the field as well as examination of herbarium specimens. Specimens were observed under a stereo dissecting microscope and measurements were taken using a ruler and a micrometer. High resolution images of the type specimen of *W.alternifolia* (held at A, acronyms according to Thiers 2022) were consulted via JSTOR Global Plants (https://about.jstor.org/). Additional specimens collected from Hainan Province (held at IBK, IBSC, KUN, and SN) and Vietnam (held at K and P) were examined through the Chinese Virtual Herbarium (https://www.cvh.ac.cn/) and the website of Muséum national d’Histoire naturelle (https://www.mnhn.fr/), respectively. Relevant taxonomic and floristic literature of *Wenchengia* was reviewed, and the terminology for morphological description follows Li and Hedge (1994) and Li et al. (2012).

## ﻿Taxonomic treatment

### ﻿ Wenchengia wui

Taxon classificationPlantaeLamialesLamiaceae

Bo Li, S.C.Xu & C.L.Xiang sp. nov.

5EBD5E6B-A64E-5F92-B035-39D4BD564BC4

urn:lsid:ipni.org:names:77365180-1

Figs 1 , 2 , 3a–e

#### Diagnosis.

This species differs from *W.alternifolia* by its opposite phyllotaxy (vs. alternate), leaves blade oblong-ovate with round to slightly truncate bases (vs. oblanceolate to lanceolate with cuneate-decurrent bases), calyces shallowly funnelform with spreading lips (vs. funnelform with straight lips), upper lip of calyx intermediately 3-lobed and the lobes ovate with acuminate apices (vs. shallowly 3-toothed and the teeth acutely deltoid), lower lip of calyx trapeziform with obliquely deltoid lobes and acute apices (vs. rectangular with rounded lobes and small tines), and nutlets longitudinally 8-ribbed (vs. 5-ribbed).

**Figure 2.**
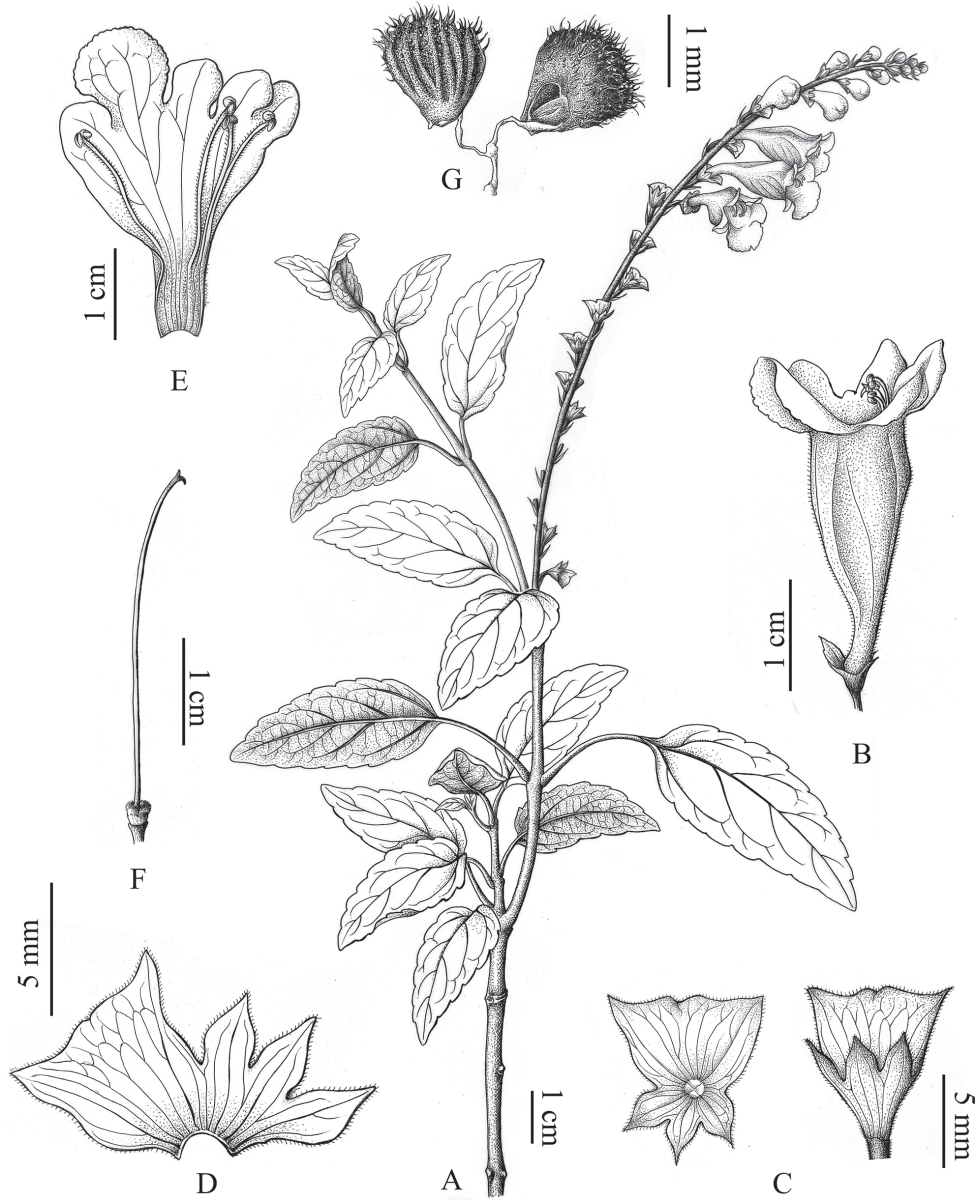
*Wenchengiawui* sp. nov. **A.** Branch with leaves and inflorescence; **B.** Flower (lateral view); **C.** Calyx (top and dorsal views); **D.** Dissected calyx; **E.** Dissected corolla, showing stamens; **F.** Ovary and style; **G.** Nutlets connected by stalks (dorsal and ventral views).

#### Type.

China • Hainan Province, Sanya City, Tianya District, Baishiling Mountain, in dry mountaintop meadows, alt. 680–740 m, 30 October 2023, *LB1118* (***holotype***: HITBC, ***isotypes***: CSH, IBSC, HIYBC, KUN, PE).

#### Description.

***Subshrubs***, 15–30 cm tall, bare at base and leafy above; young stems and the petioles, lower leaf surface, and margins of young leaves purple and hirtellous. ***Stems*** woody, flexible, prostrate or ascending, branched near base, bending into inflorescences. ***Leaves*** opposite; lamina chartaceous, oblong-ovate, 2.0–7.5 cm long, 2.0–2.7 cm wide, apex obtuse, base round or slightly truncate, margin shallowly crenate-serrate; adaxially dark green, abaxially greenish-white, primary vein convex below and slightly concave above, lateral veins 4–6 pairs; petioles 1.0–2.0 cm long. ***Inflorescence*** raceme-like, 7.0–17.5 cm long, solitary, terminal, flowers spirally arranged in bud, subsequently twisting and facing the same direction during anthesis; bracts purple, linear-lanceolate, 3.5–7.5 mm long, hirtellous; bracteoles caducous. ***Calyx*** green to purple, shallowly funnelform, 6.0–8.0 mm long, hirtellous outside, conspicuously veined; 2-lipped, lips spreading; upper lip intermediately 3-lobed, lobes equal, ovate, ca. 3.0 mm long, apex acuminate; lower lip trapeziform, 2-lobed, lobes dilated and coalescent, obliquely deltoid, ca. 6.0 mm long, apex acute. ***Corolla*** pink, tubular-campanulate, 2.8–3.7 cm long, gradually dilated into a broad throat; tube straight, 2.3–2.7 cm long, sparsely puberulent outside, bearded at lower middle inside; 2-lipped; upper lip 2-lobed, lobes subequal, ± round, 5.0–6.0 mm wide; lower lip 3-lobed, middle lobe largest, subelliptic, ca. 1 cm wide, finely crenate at margin. ***Stamens*** 4, posterior pair longer, slightly exserted from corolla tube; filaments arcuate, with capitate glandular hairs; anthers ovoid, glabrous, 2-celled, thecae divaricate. ***Style*** longer than stamens, unequally 2-clefted at apex, lobes subulate. ***Ovary*** terete, shallowly lobed, puberulent and glandular at apex, disc poorly developed. ***Nutlets*** 4, broadly obovoid, ca. 1.5 mm long, ca. 1.5 mm wide, connected to the receptacle by slender stalks; apically tuberculate and pubescent, longitudinally 8-ribbed, areole ca. 0.30 times as long as the nutlet.

#### Phenology.

Flowering from July to October and fruiting from August to December.

#### Etymology.

The specific epithet “*Wui*” is dedicated to Chengyi Wu for his tremendous contributions to the taxonomy of Lamiaceae in China, who also described and named the genus *Wenchengia* in honor of his teacher Wencheng Wu.

#### Vernacular name.

Simplified Chinese: 吴氏韫珍花; Chinese pinyin: wú shì yùn zhēn huā.

#### Distribution and habitat.

**Figure 3.**
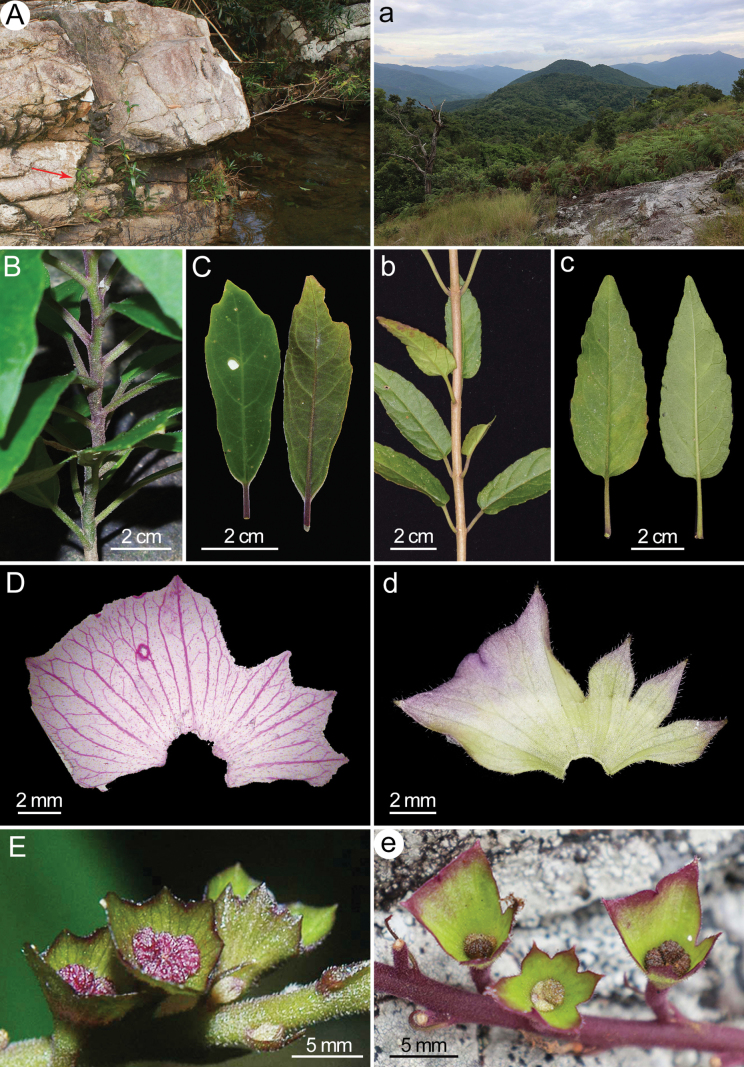
Comparison of habitats and morphology between *Wenchengiaalternifolia***(A–E)** and *W.wui* sp. nov **(a–e). A, a.** Habitat; **B, b.** Phyllotaxy; **C, c.** Leaves (adaxial and abaxial views); **D, d.** Dissected calyx; **E, e.** General morphology of calyces.

Currently, *W.wui* is only known from its type locality in Tianya District, Sanya City, in southern Hainan Province, China. It inhabits a dry mountaintop meadow, at an altitude of 680–740 m (Figs 1A, 3a). The place is surrounded by lowland rainforest and has a distinctive microclimate. It contains large, exposed rocks with sparse soil, where precipitation is abundant but poorly retained. The vegetation is predominantly ferns and interspersed with low herbs and shrubs.

#### Provisional conservation status.

*Wenchengiawui* is currently known only from the mountaintop meadows of its type locality, where it occupies a small and ecologically specialized habitat totaling less than 1 km^2^. Our field observations in October 2023 recorded fewer than 300 individuals in the extant population. Crucially, the type locality is an outdoor tourism hotspot of Sanya City, with large crowds of tourists gathering there for hiking, camping, or picnicking, causing severe disturbance and damage to the local habitat (personal observation by the authors). The site has not yet been included within any protected area, and stringent protective measures are urgently needed. Consequently, we categorize *W.wui* as Critically Endangered (CR) under criteria B [B2 a,b(ii,iii)] of the IUCN Red List Categories (IUCN 2012), for its highly restricted area of occurrence and the likely decline in both its extent and habitat quality. Precise geographic location is therefore not disclosed in the type information to prevent vandalism.

#### Taxonomic notes.

The two species of *Wenchengia* can be distinguished from each other by their phyllotaxy, leaves, calyces, and nutlets. Firstly, *W.wui* has opposite phyllotaxy (Figs 1E, 3b) whereas *W.alternifolia* has alternate phyllotaxy (Fig. 3B); the leaves of *W.wui* are oblong-ovate with round to slightly truncate bases (Figs 1D, 3c), in contrast to the oblanceolate to lanceolate leaves with cuneate-decurrent bases in *W.alternifolia* (Fig. 3C). Secondly, in *W.wui*, the calyces are shallowly funnelform with spreading lips (Figs 1K, 3e), the upper lip intermediately 3-lobed with lobes ovate and apically acuminate, while the lower lip trapeziform with lobes obliquely deltoid and apically acute (Figs 1M, 3d). In contrast, *W.alternifolia* has funnelform calyces with straight lips (Fig. 3E), the upper lip shallowly 3-toothed with teeth acutely deltoid, the lower lip rectangular with rounded lobes and very small tines (Fig. 3D). Thirdly, nutlets of *W.wui* are longitudinally 8-ribbed (Fig. 1O, P) but those of *W.alternifolia* are 5-ribbed. In addition, nutlets of *W.wui* have fragile stalks and easily detach after maturation (author’s personal observation), unlike those of *W.alternifolia*, which hang outside the calyx through a crack of the calyx tube (Li et al. 2014). Furthermore, the two species differ remarkably in their habitat. *W.wui* grows in dry, open mountaintop meadows (Fig. 1A, 3a), while *W.alternifolia* inhabits shaded, moist environment within dense rainforests (Fig. 3A).

#### Additional specimens examined.

*Wenchengiaalternifolia*: China • Hainan Province, Baoting County, Xinglong Town, Shuangximu Valley, 20 September 1935, F.C. How 73689 (HUHA00002271!, IBK00342109!, SN004110!); Hainan Province, Wanning County, Nanlin Farm, 18°43'27.45"N, 110°04'51.00"E, alt. 137 m, 22 May 2018, ZXX 18078 (KUN1437648!). Vietnam • Da Nang Province, Ba Na Hills, Mt Bani, about 25 km from Tourane, 4–13 June 1927, 3376 (P03006424!); in the same location, July 1927, 3882 (P04442939!); Thua Thien Province, Hai-Mit, 1516 (P03006423!, P03006423!).

## ﻿Discussion

With the discovery of *W.wui*, the genus *Wenchengia* is no longer monotypic, and future investigations with in-depth studies may further reveal its hidden diversity. The distinct morphology and unique habitat of *W.wui* also provide new insights into the ecological and evolutionary dynamics of *Wenchengia*. Ecologically, the divergence between *W.wui* and *W.alternifolia* may reflect fundamentally different seed dispersal mechanisms. In *W.alternifolia*, the nutlets remain suspended outside the calyx by their stalks and are dispersed by seasonal water flow, a mechanism adapted to its moist rainforest environment (Li et al. 2014). In contrast, nutlets of *W.wui* detach promptly after maturation, which is a common strategy in Lamiaceae. It is likely an adaptation to its dry mountaintop habitat, minimizing exposure to desiccating conditions. This ecological divergence reveals an intriguing link between seed dispersal mechanisms and habitat specialization in the genus.

From the evolutionary perspective, *Wenchengia* exhibits several ancestral features within Lamiaceae, including terete stems, alternate phyllotaxy, raceme-like inflorescences, and subterminal styles (Wu and Chow 1965). Its 5-lobed calyx also represents a pleiomorphic condition within Scutellarioideae, the calyces of which have evolved toward enlargement and fusion (Li et al. 2023). Furthermore, the stalked nutlet is considered as a distinct autapomorphy (Cantino and Abu-Asab 1993). These features indicate that *Wenchengia* may have a long history of independent evolution (Li et al. 2012). While showing most of the aforementioned distinctions, *W.wui* possesses opposite phyllotaxy like most Lamiaceae species, indicating that there may be some intriguing evolutionary questions underlying the new species. Consequently, *W.wui* represents an ideal model for elucidating the origin, speciation process, and historical biogeography of *Wenchengia*.

It is worth noting that *Wenchengia* warrants high conservation priority. *W.alternifolia* is already categorized as Critically Endangered (Li et al. 2014) and is listed as a Nationally Protected Plant Species (Category II) in China (National Forestry and Grassland Administration and the Ministry of Agriculture and Rural Affairs 2021). Given its extremely small population size and vulnerability to environmental disturbance, *W.wui* likewise requires urgent protection efforts, with specialized strategies based on its habitat and seed dispersal mechanism. Furthermore, ex situ conservation is strongly recommended, following the successful implementation of this approach of *W.alternifolia* (Li et al. 2014).

## Supplementary Material

XML Treatment for Wenchengia wui

## ﻿Additional information

### Author ORCIDs

Si-Cheng Xu https://orcid.org/0009-0009-1356-9929

Jin-Fei Xiao https://orcid.org/0000-0001-7729-2548

Chun-Lei Xiang https://orcid.org/0000-0001-8775-6967

AuthorName https://orcid.org/0000-0003-1628-8128

### Data availability

All of the data that support the findings of this study are available in the main text.
